# Supplementary material for: The transcriptional response to low temperature is weakly conserved across the Enterobacteriaceae
Source: mSystems. 2024 Nov 26;9(12):e00785-24. doi: 10.1128/msystems.00785-24 (PMC11651113; doi:10.1128/msystems.00785-24)
Supplement: Supplemental tables and figures — Tables S1 to S4; Figures S1 to S3. [file msystems.00785-24-s0005.pdf]

Supplementary information for Hoang and Stoebel

Table S1: Sharing of genes among the six genomes in this study

	Number of genes which are found in this many of the six genomes[†]					
Species	1	2	3	4	5	6
E. coli	981 (24.3%)	255 (6.3%)	253 (6.3%)	232 (5.7%)	483 (11.9%)	1838 (45.5%)
S. enterica	1179 (27.7%)	258 (6.1%)	237 (5.6%)	255 (6%)	483 (11.4%)	1838 (43.2%)
C. rodentium	1284 (29.1%)	278 (6.3%)	268 (6.1%)	266 (6%)	482 (10.9%)	1838 (41.6%)
E. cloacae	1695 (36%)	279 (5.9%)	177 (3.8%)	240 (5.1%)	480 (10.2%)	1838 (39%)
K. pneumoniae	1529 (34.9%)	248 (5.7%)	134 (3.1%)	163 (3.7%)	465 (10.6%)	1838 (42%)
S. marcescens	1872 (47.2%)	42 (1.1%)	35 (0.9%)	44 (1.1%)	137 (3.5%)	1838 (46.3%)

[†]For example, there are 253 genes in the genome of our *E. coli* strain that are found in a total of 3 genomes, meaning that these 253 genes are each found in *E. coli* and two other genomes. The other two genomes can vary from gene to gene.

Table S2: Growth parameters at 15°C and 37°C in LB[†]

Strain	Doubling time at 37°C (mins)	Lag time after shift to 15 °C (mins)	Doubling time at 15°C (mins)
E. coli wt	28.5 ± 0.3	63.7 ± 12.2	328.0 ± 15.2
E. coli Δ*rpoS*	25.5 ± 0.2	98.1 ± 3.6	303.5 ± 6.4
S. enterica wt	29.6 ± 1.7	96.2 ± 6.7	175.5 ± 3.8
S. enterica Δ*rpoS*	27.5 ± 1.2	97.8 ± 10.1	188.8 ± 7.4
C. rodentium wt	28.1 ± 0.6	104.0 ± 23.1	342.2 ± 30.9
E. cloacae wt	28.0 ± 3.6	90.3 ± 29.7	394.2 ± 44.6
K. pneumoniae wt	26.8 ± 0.7	119.4 ± 7.7	268.9 ± 19.8
S. marcescens wt	29.9 ± 1.3	123.8 ± 9.8	160.9 ± 7.1

[†] Values are the mean ± standard error of the mean. N = 3 for all measurements.

Table S3: Genes differentially expressed in the same direction in all six species

Locus family	*E. coli* locus tag	*E. coli* gene name	Direction of regulation
478	b3098	*yqjD*	Upregulated (higher at 15°C than 37°C)
480	b2672	*ygaM*	Upregulated (higher at 15°C than 37°C)
1120	b2528	*iscA*	Upregulated (higher at 15°C than 37°C)
2027	b0720	*gltA*	Upregulated (higher at 15°C than 37°C)
2647	b2531	*iscR*	Upregulated (higher at 15°C than 37°C)
3056	b2905	*gcvT*	Upregulated (higher at 15°C than 37°C)
3422	b0946	*zapC*	Upregulated (higher at 15°C than 37°C)
3458	b1060	*bssS*	Upregulated (higher at 15°C than 37°C)
3871	b0189	*rof*	Upregulated (higher at 15°C than 37°C)
4196	b3728	*pstS*	Upregulated (higher at 15°C than 37°C)
2124	b0401	*brnQ*	Downregulated (lower at 15°C than 37°C)
3753	b2324	*mnmC*	Downregulated (lower at 15°C than 37°C)
3837	b2565	*recO*	Downregulated (lower at 15°C than 37°C)
3933	b0153	*fhuB*	Downregulated (lower at 15°C than 37°C)
4108	b3409	*feoB*	Downregulated (lower at 15°C than 37°C)
4203	b4557	*yidD*	Downregulated (lower at 15°C than 37°C)

Table S4: Strains used in this study

Species	Published strain number	Lab isolate number (if different)	Genotype	Source	Reference	NCBI assembly used for RNA-seq analysis
E. coli	BW27786	DMS2537	F-, Δ(araD-araB)567, ΔlacZ4787(::rrnB-3), λ-, Δ(araH-araF)570(::FRT), ΔaraEp-532::FRT, φPcp13araE534, Δ(rhaD-rhaB)568, hsdR514	Lab collection	doi: 10.1099/00221287-147-12-3241	GCF_000005845.2
E. coli	DMS2545		ΔrpoS746::kan derivative of DMS2537 (F-, Δ(araD-araB)567, ΔlacZ4787(::rrnB-3), λ-, Δ(araH-araF)570(::FRT), ΔaraEp-532::FRT, φPcp13araE534, Δ(rhaD-rhaB)568, hsdR514, ΔrpoS746::kan)	Lab collection	doi: 10.1128/jb.00755-16	GCF_000005845.2
S. enterica	14028s	DMS2709	wild-type	Salmonella Genetic Stock Center		GCF_000022165.1
S. enterica		DMS3189	Δ*rpoS*::cm derivative of 14028s	McClelland Lab		GCF_000022165.1
C. rodentium	DBS100	DMS2868	wild-type	ATCC		GCF_000027085.1
E. cloacae	ATCC13047	DMS2862	wild-type	ATCC		GCF_000025565.1
K. pneumoniae	MKP103	DMS2797	Strain KPNIH01, Δ*KPC-3::FRT*	Manoil Lab	doi: 10.1128/JB.00352-17	GCF_000281535.2
S. marcescens	Db11	DMS2843	wild-type	*C. elegans* Stock Center		GCF_000513215.1

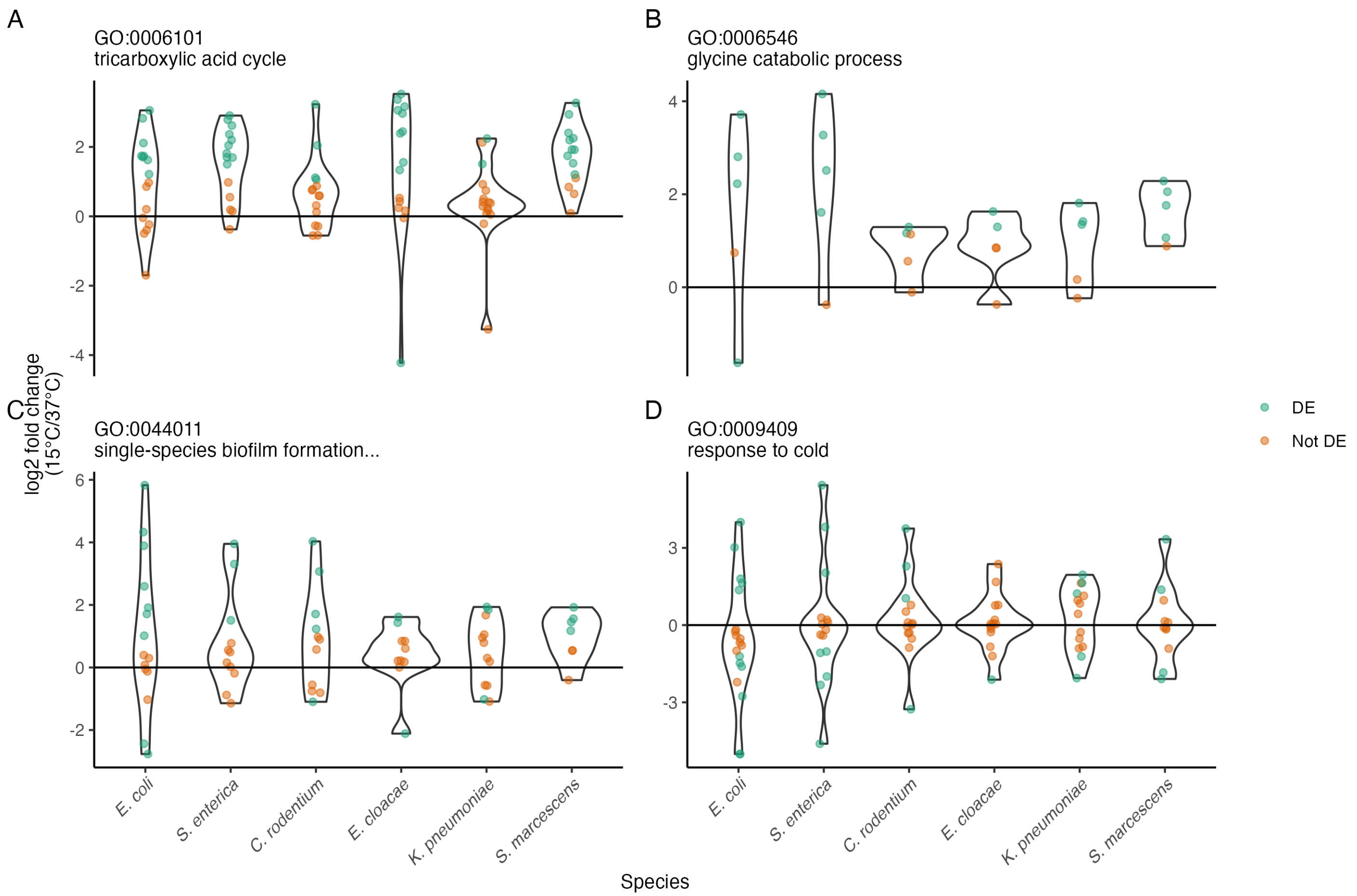

Figure S1: Groups of genes that change in multiple species. Log_2-fold change of gene expression between 15°C and 37°C for genes annotated as involved in (A) the tricarboxylic acid cycle, (B) glycine catabolism, (C) single-species biofilm formation on an inanimate surface, and (D) response to cold. Green dots represent a single DE gene, while orange dots are a gene that is not DE. Violin plots show the distributions of log_2-fold changes of the individual genes.

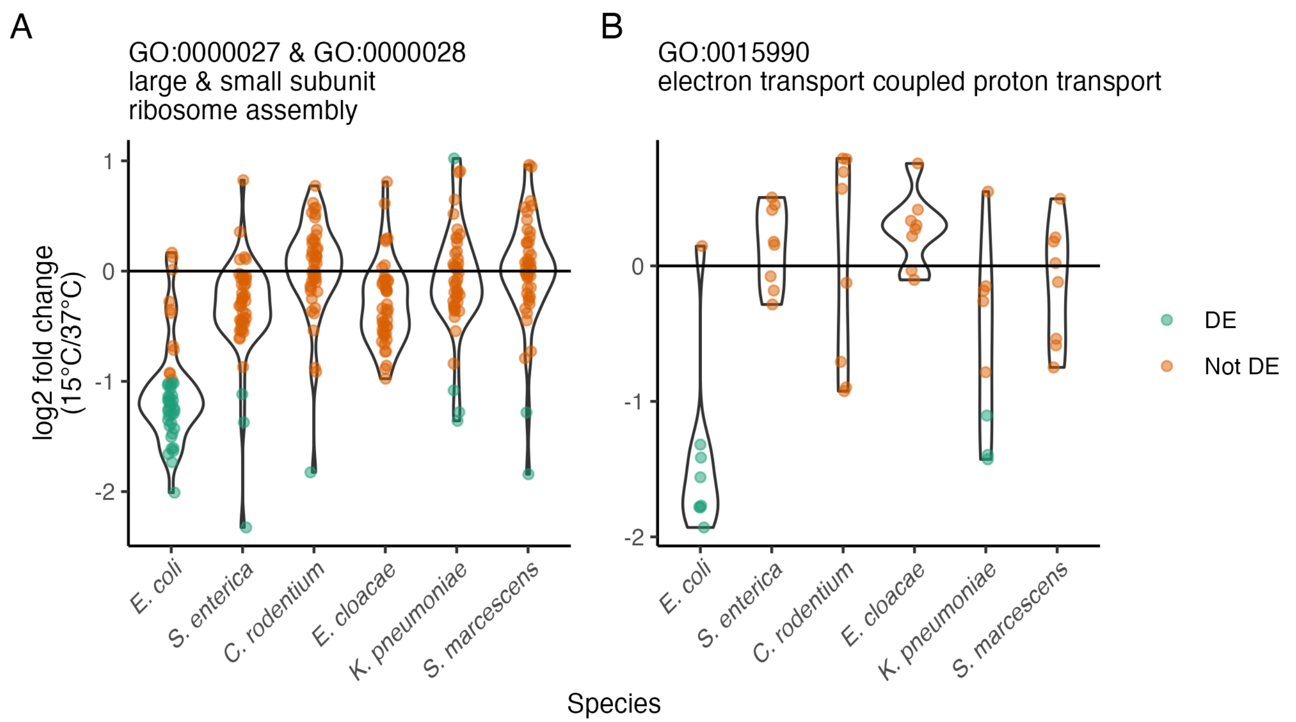

Figure S2: Responses to low temperature unique to *E. coli*. Log$_2$-fold change of gene expression between 15°C and 37°C for genes annotated as involved in (A) production of the ribosome, and (B) electron transport coupled proton transport. Green dots represent a single DE gene, while orange dots are a gene that is not DE. Violin plots show the distributions of log$_2$-fold changes of the individual genes.

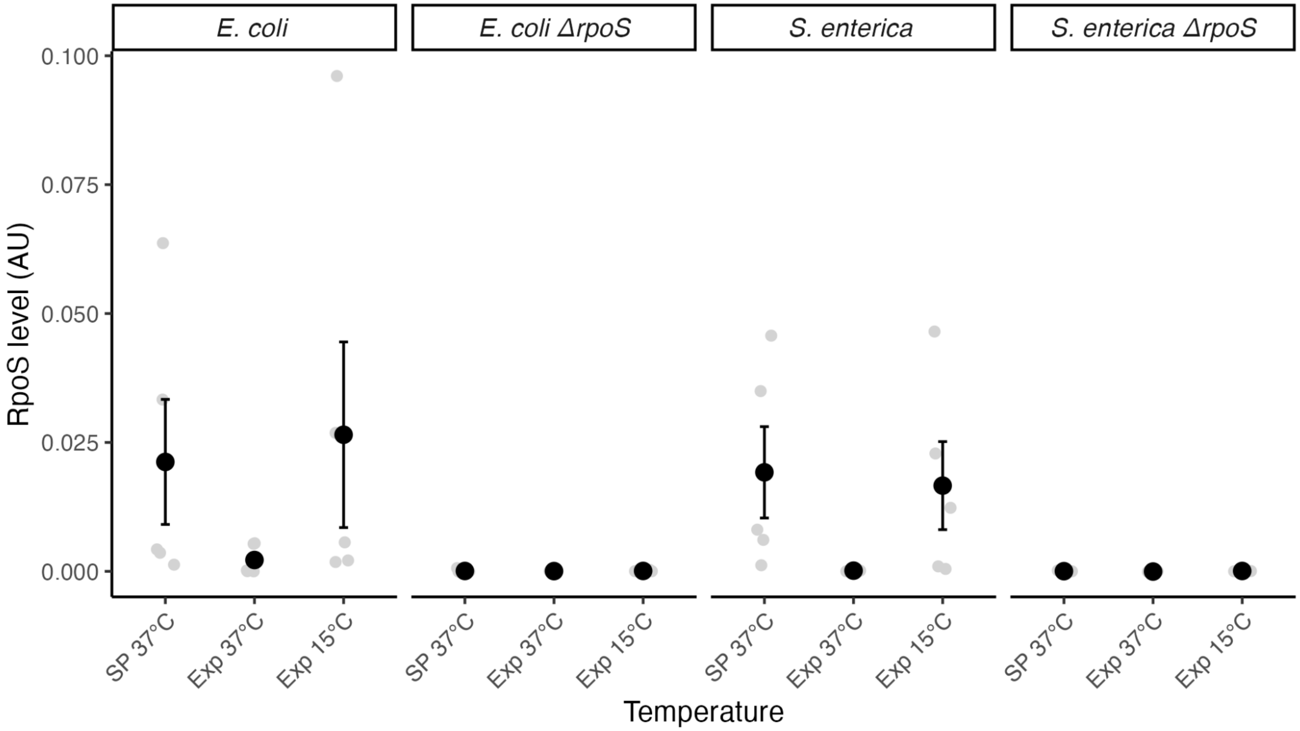

Figure S3: RpoS levels as measured by Western blotting in stationary phase (SP), exponentially growing cultures at 37°C (Exp 37°C), or of those same cultures 3 hours after a shift to 15°C (Exp 15°C). Light gray dots are individual replicates, black dots are the mean, and error bars are the standard error of the mean.
